# Severe Malaria: A Case of a Significant Rapid Rise in the Parasite Level

**DOI:** 10.1155/2020/5206186

**Published:** 2020-03-16

**Authors:** Muhammed Atere, Lloyd Muzangwa, Foma Munoh Kenne, Cherry Hanna, Jessie Saverimuttu, Virginia Kopetz

**Affiliations:** Richmond University Medical Center, New York City, NY, USA

## Abstract

Malaria is transmitted by the *Plasmodium* parasite, and most of the cases reported in the United States are often as a result of patients with recent return from endemic areas. Prompt diagnosis and treatment, particularly if there is severe parasitemia and drug failure, is essential in preventing mortality. Our patient had an unusual rapid rise in parasite but susceptible to intravenous artesunate.

## 1. Introduction

Malaria is a major cause of morbidity and mortality in the developing countries [1, 2]. However, most of the rare cases seen in the United States are often as a result of travelers entering the country after visiting endemic areas [3]. Rapid rise in parasitemia level within a short duration may not be anticipated, but early administration of an effective drug is important to reduce mortality. Exchange blood transfusion has been suggested for the treatment of patients with severe malaria and high parasitemia [4]. For early diagnosis, it is paramount to consider malaria in every febrile patient with a history of travel to an area endemic for malaria [4]. The purpose of this case report is to emphasize an unusual rise in parasitemia. We report a case of severe malaria manifested by a significant rise in parasitemia resistant to atovaquone and proguanil but susceptible to intravenous artesunate.

## 2. Case Report

**Figure 1:**
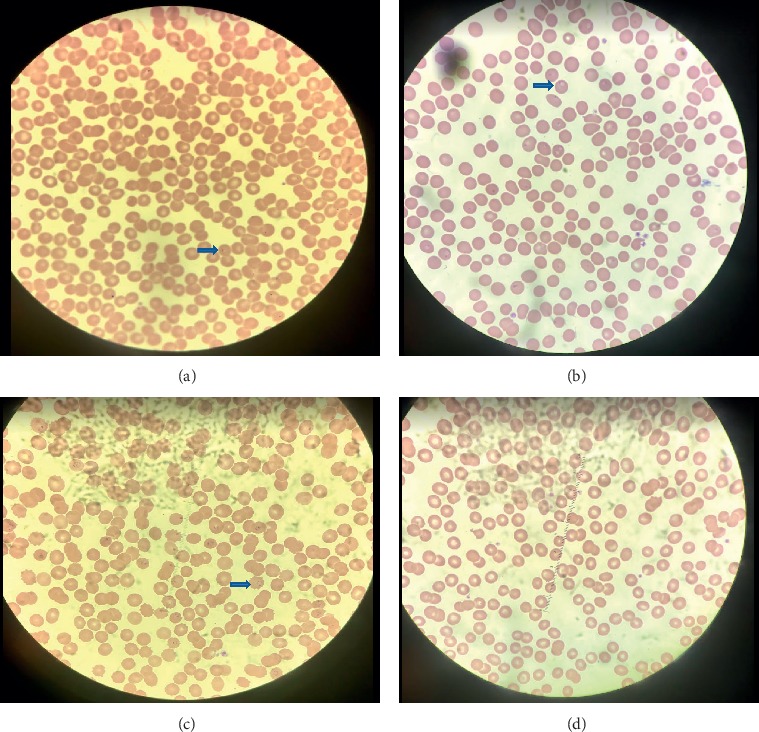
Thin peripheral blood smear: blue arrow showing signet-ring of *Plasmodium falciparum* in a red blood cell; (a) parasitemia level of 1.6%, (b) parasitemia level of 7.6%, (c) parasitemia level of 12.24%, (d) parasitemia level of 0.1%.

A 46-year-old female without a significant past medical history presented to the emergency room because of fever, myalgias, and body aches for past two days. She denied cough, dysuria, diarrhea, convulsions, and sick contact. However, she recently returned from Nigeria 7 days before presentation. Her initial vital signs were within the normal limit, but she spiked a fever of 101.7 F later on the same day of presentation. Physical examination was normal. She was alert and oriented to time, place, and person. She was ambulatory and was anicteric. Initial white blood cell count (6,000/*μ*l) and hemoglobin (13 g/dl) levels were within normal limits; however, platelet count was low (83,000/*μ*L). A comprehensive metabolic panel was normal with a random blood glucose of 136 mg/dl, creatinine of 1.1 mg/dl, and bicarbonate level of 25 mmoL/L, and a hepatic panel showed mild elevation in total bilirubin (1.2 mg/dL), aspartate transaminase (43 U/L), and alanine transaminase (59 U/L). Urinalysis was negative for blood, and red blood cell was 0–3/HPF. A chest X-ray was negative for an acute pulmonary mass, infiltrate, edema, effusion, or pneumothorax. She had a peripheral blood smear (Figure 1). Microbiology revealed a parasitemia level of 1.6% of *Plasmodium falciparum* at 1437 hours on Day 0. Despite being on the full dose of oral atovaquone and proguanil, the parasitemia level continued to rise from 1.6% to 7.6% at 0839 hours on Day 1 and then to 12.24% at 1154 hours on Day 2 with persistent intermittent fever spikes. The Center for Disease Control and Prevention (CDC) was contacted, and the patient was supplied with three doses of intravenous artesunate at 2.4 mg/kg per dose. After the second dose, the parasitemia level was at 0.1% at 0458 hours on Day 3, and after the third dose it was negative. A repeat test also was negative. The patient continued to be on atovaquone and proguanil for 3 more days. The patient's symptoms resolved, and she stopped spiking fever and platelets count improved to 229,000/*μ*L. She was discharged with scheduled repeat lab work weekly for 4 weeks as an outpatient.

## 3. Discussion

Many US clinicians and laboratory personnel are unfamiliar with the diagnosis and treatment of malaria [5]. Malaria remains a diagnostic and treatment challenge for US clinicians as increasing numbers of persons travel to and emigrate from malaria-endemic areas. A strong evidence base exists to help clinicians rapidly initiate appropriate therapy and minimize the major mortality and morbidity burdens caused by this disease [5].

More than ten manifestations define severe malaria, and among them is a parasite level of more than 10% [6]. This usually requires intravenous treatment with antimalarial drugs which may not be available in some hospitals in the United States, resulting in a delay in treatment. In the treatment of severe malaria, intravenous artesunate is more rapidly acting than intravenous quinine in terms of parasite clearance, is safer, and is simpler to administer, but whether it can reduce mortality is uncertain [7, 8]. Recent studies have shown that artesunate is an effective drug for treating severe malaria [7–9]. The level of *Plasmodium falciparum* parasitemia at clinical presentation has repeatedly been shown to correlate with severity of disease, which was the only criterion that qualified as a case of severe malaria [10]. The parasite percent was calculated using the formula: number of parasitized red blood cells/total red blood cells counted in 25 fields × 100. Our patient initially had a parasitemia level of 1.6% with 8 times increase within 48 hours despite a full course of oral atovaquone and proguanil. One possibility may have been treatment failure from possible poor oral absorption from sequestered malaria parasites in the gastrointestinal tract. A rise in parasitemia level after treatment with artemisinin-based drugs and quinine has been documented, which may double but not as high as 8 times as seen in our patient [11, 12]. She received intravenous artesunate supplied promptly by the CDC. By the second dose of artesunate, the parasitemia had dropped to 0.1% and completely cleared after the third dose, which improved her symptoms and platelets.

### 3.1. Interpretation

It is reasonable for artesunate to become the treatment of choice for severe *falciparum* malaria in adults. Parasitemia at clinical presentation can be used to correlate with severity of disease.
